# fMRI Food Cue Reactivity as a Predictor for BMI Change Following Roux-en-Y Gastric Bypass (RYGB) or Diet Intervention

**DOI:** 10.17756/jocd.2021-043

**Published:** 2021-06-28

**Authors:** Shaunte Baboumian, Carol Cheney, Sumiyah Enayet, Spiro P. Pantazatos, Allan Geliebter

**Affiliations:** 1 Icahn School of Medicine at Mt Sinai, New York, NY, USA; 2 University of North Carolina, Chapel Hill, NC, USA; 3 Columbia University Irving Medical Center, New York, NY, USA

**Keywords:** Obesity, fMRI, Bariatric surgery, RYGB, Dietary restriction, BMI, Cue reactivity, Clinically severe obesity (CSO), Region of interest (ROI)

## Abstract

Prevalence of severe obesity continues to increase, with only bariatric surgery showing long-term efficacy for sustained weight loss. Individuals with severe obesity (vs normal weight) show greater fMRI responsivity to high energy dense (ED) vs low ED food cues and reduced responsivity post-surgery. We examined responsivity to high vs low ED cues pre-intervention in association with postsurgical (RYGB) or dietary weight-loss (dWL) change in BMI at 4 and 18 mo. Region of interest (ROI) analysis employed separate ANCOVA models; group as single factor with three levels and baseline activation and interaction with group covarying for age and gender as nuisance covariates. Significant results were identified at p < 0.1 false discovery rate (FDR) corrected, following multiple comparisons across ROIs. In the precentral gyrus (motor and motor readiness area), higher baseline activation was associated with greater %BMI reduction in RYGB at 4 and 18 mo and less %BMI reduction in dWL at 4 mo (p = 0.006 uncorrected, P < 0.1 FDR corrected). The findings show opposite directionality in predicting change in BMI for RYGB vs. dWL from responsivity to high vs low ED food cues in the precentral gyrus. Greater baseline motor planning to ingest high ED foods may be associated with reduced weight loss in dWL, and with greater weight loss in RYGB due to neuromodulatory effects of surgery.

## Introduction

According to the Centers for Disease Control and Prevention, the prevalence of obesity in the US was 42.4% in 2018 [1]. Currently, bariatric surgery is the only treatment for obesity with established long-term efficacy [2, 3]. There are still challenges, however, and a significant proportion of patients are not successful, defined as losing less than 50% of excess body weight [4].

Roux-en-Y gastric bypass (RYGB) and sleeve gastrectomy (SG) are the most common bariatric procedures [5]. In RYGB, a small pouch of the stomach is created and connected to the mid-jejunum, and the rest of the stomach as well as the duodenum and proximal jejunum are bypassed. RYGB surgery leads to 69% loss of excess BMI after two years [6]. Studies suggest that neither malabsorption nor dumping syndrome can account for the degree of weight loss associated with RYGB [7]. For instance, studies show that indicators of clinical malabsorption such as reductions in albumin and prealbumin, and increases in fecal fat, are not observed after RYGB [8]. Furthermore, no significant correlation was found between severity of dumping syndrome symptoms and weight loss after RYGB [5].

One possibility is that changes in neural responsivity to food cues help mediate surgical weight loss. Animal studies show that motivated behavior to consume food is controlled by several brain areas, including the hypothalamus and mesolimbic reward pathway (ventral tegmental area (VTA), amygdala, hippocampus, and ventral striatum) [9]. fMRI studies show that individuals with obesity (vs. normal weight) are more responsive to highly palatable food cues [10, 11]. Additionally, bariatric surgery appears to attenuate palatable food cue reactivity, potentially contributing to postsurgical weight loss [12].

In this study, we compared RYGB patients to a dietary weight loss (dWL) group and a nonintervention group as controls. We used a region of interest (ROI) fMRI approach. Based on prior literature that higher activation to palatable stimuli in certain brain regions is correlated with obesity [10], we anticipated that individuals would show a correlation between baseline activation to high ED (vs. low ED) food stimuli and subsequent % reduction in BMI due to neuromodulatory effects of the surgery. In the RYGB group, we expected that the effects of surgery would decrease the activation to high ED foods to a low level for all individuals, but this decrease would be more marked for those with higher baseline reactivity, leading to greater post-surgical weight loss. Whereas in the dWL (nonsurgical dietary weight loss) group, we hypothesized that higher baseline activation to high ED (vs. low ED) food stimuli would be associated with smaller % change in BMI because the baseline high ED responsivity would inhibit weight loss. In the current study, we assessed baseline neural activation to high ED (vs low ED) food images in participants receiving RYGB surgery, undergoing a dietary low-calorie intervention (dWL) or receiving no treatment (NT). We then tested whether baseline activation correlated with change in BMI at 4 mo and 18 mo post intervention among the groups.

## Methods

### Study participants

Seventeen RYGB surgery participants were recruited from the Center for Weight Loss Surgery at a large university-affiliated hospital in New York City. Additionally, matched groups of participants were recruited from the same hospital catchment area who committed to not undergo surgery or any weight loss interventions outside the scope of the study for 22 mo. One group underwent a 12-week dietary weight loss (dWL) program, and another group received no treatment (NT). 47 participants (17 RYGB, 14 dWL, and 16 NT) had follow up data at 4 mo, and 20 (5 RYGB, 10 dWL, and 5 NT) at 18 mo.

dWL participants were instructed to consume an all-liquid low-calorie diet (LCD) consisting of 925 kcal/day from 5 ProCal (100 g) packets (R-Kane, Piscataway, N.J.) and sugar-free fiber (Metamucil) supplements provided to them, together with 3.5 cups of 1% milk. The 3-month dietary weight loss program included weekly nutritional counseling and cognitive behavioral therapy. The subject characteristics are shown in Table 1. ANOVA was used to test for group differences.

Exclusion criteria were: BMI < 40.0 or > 55.0; left handedness; pregnancy (serum test), lactation, planning to become pregnant in next 18 mo, or < 1 yr postpartum; smoking or recent (previous 3 mo) smoking cessation; consumption of ≥ 3 alcoholic beverages/d; meeting DSM criteria for substance use disorder; current suicidal ideation; history of severe head injury, neurological disorder, psychotic or bipolar disorder, major depression, or hospitalization for psychiatric illness within the past year (may affect brain activation); history of significant health problems, such as cancer, or heart, kidney, thyroid, gastrointestinal, and liver disease; diabetes; uncontrolled hypertension (>140/90 mmHg); daytime sleepiness based on a score >12 on the Epworth sleepiness scale (to avoid a likelihood of falling asleep in the scanner); previous abdominal or brain surgery; known claustrophobia for an enclosure, such as the MRI scanner; maximum supine abdominal width of > 65 cm or sagittal diameter of > 50 cm (the scanner bore diameter is 70 cm, and the vertical distance above the table is 55 cm); body wt > 550 lbs (scanner table capacity is 550 lbs); metal implants, non-removable metal dental retainers or pacemakers.

### Design and procedure

The fMRI scans were acquired at 1-month pre-RYGB or dWL, and body weights were measured at approximately 4 mo and 18 mo. Participants consumed a 250 kcal nutritionally complete liquid meal (Glytrol; Nestlé Nutrition, Vevey, Switzerland) 12 h prior to the scan. After an overnight (12 h) fast, participants reported to the fMRI Research Center at the Columbia University Irving Medical Center between 11 AM and 1 PM. Participants ingested an additional 250 kcal of Glytrol 1.5 h prior to the scan to control their prescan food intake and minimize subject differences in baseline hunger and fullness ratings, which were recorded based on visual analog scales (0-100).

During the fMRI scan, participants were presented with visual and auditory representations (cues) of high ED foods (e.g., pepperoni pizza, fudge sundae), low ED foods (e.g., raw vegetables, fruits) and neutral nonfoods (office supplies; e.g., pencil, stapler). Auditory data were not analyzed for the purposes of this study. The visual cues were transmitted through eye goggles. In each block, 10 consecutive images were presented for 4-sec each (total block duration 40 sec), with a 52-sec pre block baseline epoch and a 40-sec post block epoch. The first 12 sec of each run were attained for purposes of magnetic equilibration and were not included in the analysis. Each run contained one block. There were 2 similar but not identical runs per cue condition, for a total of 6 runs (blocks) [10].

### Imaging acquisition and analyses

A 1.5-T twin-speed fMRI scanner (GE Healthcare Technologies, Waukesha, Wisconsin) with quadrature RF head coil and 70 cm bore diameter was used. Passive restraints included head pads and tape across the forehead to minimize motion. Three-plane localization was used to verify head position. In each run, 36 axial scans of the whole brain were acquired, with each scan consisting of 25 contiguous slices (4 mm thick), with a 19 × 19 cm field of view, an acquisition matrix size of 128 × 128, and 1.5 mm × 1.5 mm in plane resolution. The axial slices were parallel to the AC/PC line. T2*-weighted images with a gradient echo pulse sequence (echo time = 60 ms, repetition time = 4 seconds, flip angle = 60-degree angle) were acquired with matched anatomic high resolution T1*-weighted scans.

Functional data were analyzed with SPM8. Before statistical analyses, the realigned T2*-weighted volumes were slicetime corrected, spatially transformed to a standardized brain (Montreal Neurologic Institute) and smoothed with an 8-mm full-width half-maximum Gaussian kernel. The first 3 volumes were discarded. First-level regressors were created by convolving the onset of each blocked condition (visual high ED, low ED, and nonfood) with the canonical HRF with duration of 40 seconds. Second-level analyses used contrast estimates from the 1^st^ level pre-surgery sessions in a 1-way ANCOVA, first factor being subject group, with nuisance covariates age and gender. The covariate of interest, percent change in BMI (post-baseline at 4 mo or 18 mo, depending on the model, and the interaction with the subject group was included in the SPM models, but only the nuisance covariates (age and gender) and grand mean were removed when extracting average signal in each ROI using Marsbar. The extracted ROI signals (contrasts of parameter estimates) were stored in a spreadsheet for the main ROI analyses using R Studio 4.0.3. In R, separate ANCOVA models (group as single factor with three levels and baseline activation and interaction with group) were estimated for each ROI. The p-values for the 14 ROI x Group interaction terms were corrected for multiple comparisons across ROIs and contrasts, using the false discovery rate (FDR) to identify significant results at p < 0.1 FDR corrected, consistent with thresholds used in the literature [13] to lower the rate of false negatives.

Effects of age, sex, or hunger rating on BMI change were not detected, and thus not included in the below models. In addition, age and sex were regressed (removed) from BOLD responses prior to the ROI analyses. Significant Group x Activation interactions were followed by post-hoc contrasts to compare regression lines between groups.

The models and graphs in R were generated using the following lines of code:

>>aov(Percent_Change_BMI~0 + ROI_RESPON-SE+Group+ROI_RESPONSE:Group)

>>Anova(modl, type = “III”)

>>summary.lm(modl)

>>plot_model(modl, type = “pred”, show.data = 1, terms = c(“ROI_RESPONSE”, “Group”)

ROI_RESPONSE refers to the contrast of parameter estimates (i.e. activation) for each brain region. Two separate models were run for 4 mo and 18 mo since fewer subjects were available at 18 mo. For purposes of this study, the contrast high ED > low ED was used to index brain activation, and nonfood stimuli were not included in the contrast.

## ROI Selection

We designated ROIs based on prior research from our group that found reductions in brain region activity after RYGB surgery in response to visual cues [12], and from a similarly designed study that investigated a nonsurgical dietary weight loss intervention [14]. Reward-related ROIs included: anterior cingulate (ACC), frontal operculum, lentiform nucleus, middle cingulate cortex, medial frontal gyrus, insula, and nucleus accumbens. Motor and motor planning ROIs included the precentral gyrus and superior frontal gyrus (SFG). An inhibitory area included middle frontal gyrus, and attention-related ROIs included inferior parietal lobule, precuneus, and posterior cingulate [15] (see Table S1 for the selected ROI coordinates).

ROI masks consisted of 10 mm spheres built around coordinates based on peak voxels in the clusters reported in the above studies. The value for each ROI consisted of contrast of parameter estimates (high ED > low ED) averaged across all voxels within each ROI. The values were extracted from the 2^nd^ level SPM model mentioned above using Marsbar 0.44 (http://marsbar.sourceforge.net).

## Results

### Demographic and anthropometric data

There were no significant differences between the groups for age (p = 0.39) or for baseline BMI (p = 0.075) (Table 1). As expected, the RYGB group showed a greater reduction in BMI (BMI change = −20.6% ± 3.6% SD) than the other groups (dWL and NT) at 4 mo (p < 0.001) and at 18 mo (BMI change = −38.1% ± 9.1%, p < 0.001). The dWL group showed a reduction in BMI from baseline to 4 mo (BMI change = −12.3% ± 2.3%, p = 0.001), but not from baseline to 18 mo (BMI change = 0.0% ± 7.9%) (p = 0.91). The NT group had no significant change in BMI at 4 mo (BMI change = −1.8% ± 2.7%, p = 0.38) or 18 mo (BMI change = −3.0% ± 12.1%, p = 0.58).

### 4 mo % change in BMI in relation to baseline ROI activation

**Figure 1.**
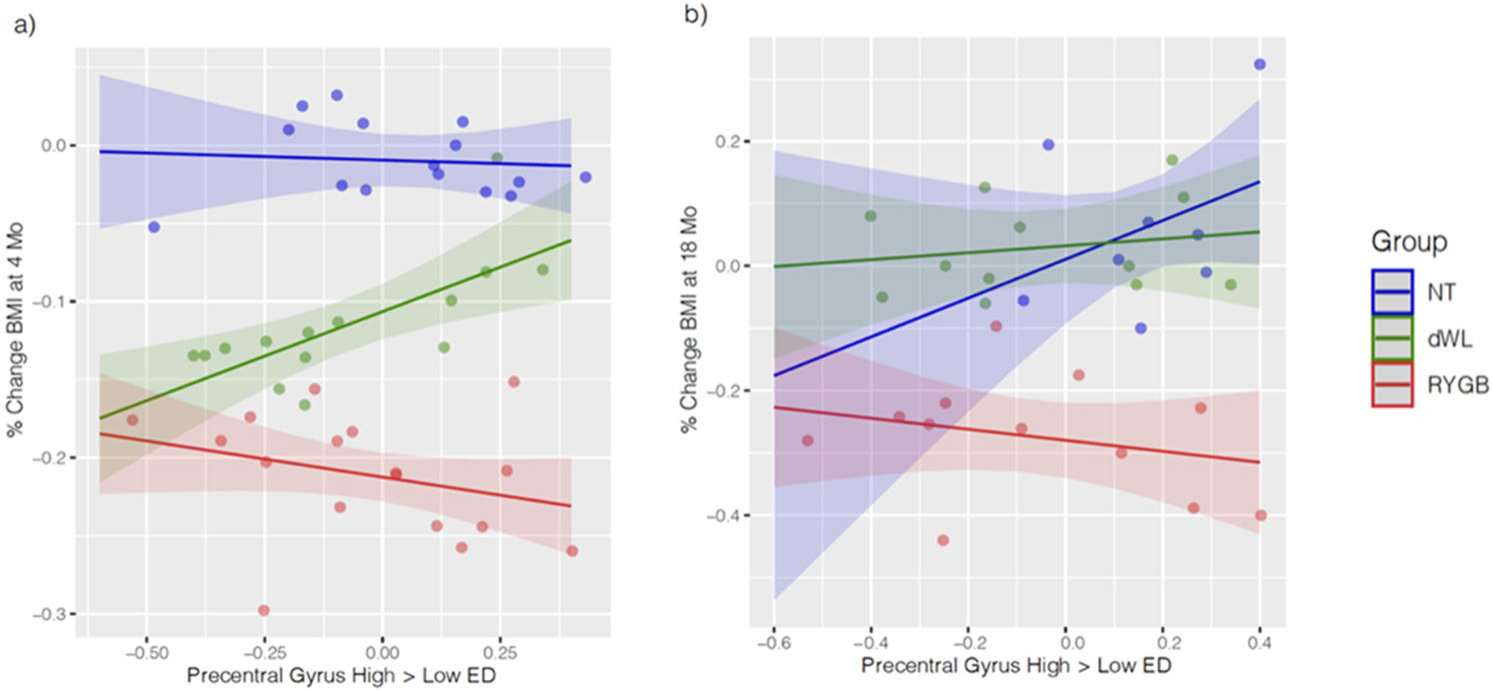
ROI activation for high energy dense (ED) > low energy dense (ED) food cues in relation to % change BMI at 4 mo and 18 mo. **a) precentral gyrus (PCG) 4 mo.** There was a significant interaction between group and PCG in predicting BMI change (p=0.006 uncorrected, p<0.1 FDR, see Table 2). There was a negative slope within RYGB such that greater activation was associated with a greater reduction in BMI and a positive slope within dWL such that greater activation was associated with less reduction in BMI (see Table 3). Shaded regions indicate 89% high density intervals (HDI) (see http://www.strengejacke.de/sjPlot/reference/plot_model.html). **b) precentral gyrus (PCG) 18 mo.** The interaction between group and PCG in predicting BMI change was not significant (see Table 2). There was a negative slope within RYGB such that greater activation was associated with a greater reduction in BMI and a positive slope (ns) within dWL such that greater activation was associated with less reduction in BMI (see Table 3). Shaded regions indicate 89% high density intervals (HDI).

There was a significant difference in the association between baseline activation and weight loss at 4 mo between RYGB, dWL and NT groups (Group x Activation interaction, p = 0.00628 uncorrected, p < 0.1 FDR (Figure 1a and Table 2). In the RYGB group, greater activation of high ED (vs. low ED) food stimuli in the precentral gyrus (PCG), MNI coordinates [−48, −2, 28], was associated with greater % change in BMI, t = −3.32, p =0.002 uncorrected (Figure 1a and Table 3). In the dWL group, more activation in the PCG correlated with a smaller % change in BMI at 4 mo, t = 3.1, p = 0.003, uncorrected (Figure 1a and Table 3).

### 18 mo % change in BMI in relation to baseline ROI activation

There was no significant overall Group x Activation interaction in the precentral gyrus in predicting % change in BMI at 18 mo, p = 0.295, ns (Figure 1b and Table 2). However, the direction of the correlations were consistent with those in the 4 mo analysis: baseline activation positively correlated with % change in BMI within the RYGB group, beta parameter estimate = −0.14, p = 0.39, ns, and negatively correlated with % change in BMI in the dWL group, beta parameter estimate = 0.06, p = 0.66, ns (Figure 1b and Table 3).

## Discussion

This study examined neural activation in response to high ED vs. low ED visual food cues based on ROI analyses and BMI change at 4 and 18 mo among participants undergoing weight loss through surgery (RYGB), diet (dWL), or no treatment (NT). As expected, the RYGB group showed a greater reduction in BMI than the dWL and NT groups at 4 and 18 mo. The dWL group significantly reduced their BMI from baseline to 4 mo, but not from baseline to 18 mo, given that the formula diet ended by 3 mo, with subsequent weight regain. Also, as expected, the NT group did not show a change in BMI at either 4 or 18 mo.

At 4 mo, the baseline activity in the precentral gyrus (primary motor area and motor readiness area) showed opposite correlations with weight loss in the RYGB and dWL groups. More baseline activity to high ED vs. low ED food cues in the precentral gyrus was associated with greater weight loss for the RYGB group at 4 mo, whereas more baseline activity predicted less weight loss for the dWL group. In a previous study from our group, the precentral gyrus was shown to be activated in response to high ED food stimuli in individuals with obesity and binge eating [16]. We speculate that this higher neural reactivity in response to high ED foods reflects an increased motor readiness for food, which accounts for the correlation with greater reduction in BMI in the RYGB group (vs. the dWL group). The motor readiness for high ED food may have been reduced to a low level across all surgical participants due to the neuromodulatory effects of bariatric surgery (indeed, we previously found reduced activation to high ED food cues <1 mo post RYGB in the precentral gyrus [17]. Those with a higher baseline motor readiness would have had a more marked reduction and thus greater weight loss. At 18 mo, similar to the 4 mo findings, in the precentral gyrus, RYGB showed greater activation with greater % change in BMI, opposite in direction to dWL, although the interaction between groups in predicting BMI change was not significant. This may be due to the smaller sample size at 18 mo due to dropouts.

Another study examined neural predictors in bariatric sleeve gastrectomy surgery (SG), comparing brain activations to palatable food stimuli at baseline to weight loss 12 mo post op. Increased fMRI activity in the nucleus accumbens, a reward area, predicted less weight loss at 12 mo [18]. We did not observe significant predictive effects of brain activation in the nucleus accumbens or other reward-related areas on weight loss. This may be related to the different bariatric operations studied, SG vs. RYGB, which show some differences in brain responsivity post-surgery [19, 20]. Based on our previous studies [12, 19] that show reduced activation in reward-related areas in response to high ED food stimuli, we anticipated that greater baseline activation in these areas would correlate with greater weight loss. That we did not observe this, but found evidence only for PCG activity, suggests that motor readiness or planning to ingest food, may have a greater impact on actual food intake than liking a food (reward), and is a better predictor of weight loss in response to surgical or dietary intervention.

There are several limitations of our study, including a small sample size and lack of randomization into groups, as this is difficult with surgical interventions. It is also possible that the bariatric surgery candidates had a lower socioeconomic status (SES) than the otherwise matched controls because many surgeries were covered by Medicaid. The samples were also not gender-balanced, although gender was adjusted for in the imaging analyses. Females are overrepresented in bariatric surgery and therefore they were also overrepresented in our matched control groups. We also did not control for menstrual cycle for the female participants, which may affect cue-reactivity activation [21]. Strengths of our study include a within-subjects longitudinal design, which allows for detection of relatively small effects.

## Conclusions

Baseline fMRI activation in the precentral gyrus in response to high vs low ED food stimuli was correlated with future weight change at 4 mo, depending on the weight loss intervention. Higher activity in this motor region led to greater reduction in BMI in the RYGB group, and smaller reduction in BMI in the dWL group. A similar pattern was observed at 18 mo, although the interaction between RYGB and dWL was not significant. We speculate that greater activity reflects motor planning for food consumption, which may impede weight loss in the dWL group. The neuromodulation by surgery may act to reverse the effect and help decrease the consumption of such food, with a greater impact on those who initially exhibited higher neural responsivity.

## Supplementary Material

Supplementary

## Figures and Tables

**Table 1.** Participant Demographics, % BMI Change, and Appetite Ratings

Group	RYGB (n=17)	dWL (n=14)	NT (n=16)	p value^b^
Age	38.4 ± 10.3	39.1 ± 9.6	34.2 ± 11.8	.388
Sex	f=16; m=1	f=10; m=4	f=14; m=2	.209
Baseline BMI (kg/m^2^)	44.3 ± 3.9	42.7 ± 4.0	41.2 ± 3.4	.075
% Change BMI @ 4 mo	−20.6% ± 3.6%	−12.3% ± 2.3%	−1.8% ± 2.7%	.001*
% Change BMI @ 18 mo ^a^	−38.1% ± 9.1%	0.0% ± 7.9%	−3.0% ± 12.1%	.000*
Baseline Prescan fullness ratings	48.1 ± 36.8	43.9 ± 32.3	44.8 ± 25.5	.929
Baseline Prescan hunger ratings	34.6 ± 25.3	41.9 ± 29.7	32.0 ± 33.0	.646

b from ANOVA

* p<0.01

a At 18 mo, RYGB (n = 5), dWL (n = 10), NT (n = 5)

**Table 2.** ANCOVA Analyses for Precentral Gyrus Across Groups (RYGB, dWL, NT)

	Sum Sq	Df	F value	P value
**4 Months**				
Precentral Gyrus - High > Low ED	0.01025	1	9.8504	0.003185**
Group	0.8992	3	288.0842	< 2.2E-16***
Precentral Gyrus - High > Low ED X Group	0.01201	2	5.771	0.00628**
Residuals	0.04162	40		
**18 Months**				
Precentral Gyrus - High > Low ED	0.00213	1	0.1999	0.6585
Group	0.89842	3	28.1543	2.52E-08***
Precentral Gyrus High > Low ED X Group	0.02723	2	1.2799	0.295
Residuals	0.27656	26		

** p<0.01

*** p<0.001

**Table 3.** Precentral Gyrus ANCOVA estimates for model coefficients and associated t and post-hoc p-values

	Estimate	Std. Error	t-value	P value
**4 Months**				
Precentral Gyrus - High > Low ED	0.113908	0.036294	3.139	0.00318**
RYGB	−0.212441	0.00789	−26.924	< 2E-16***
dWL	−0.106492	0.009063	−11.75	1.51E-14***
NT	−0.009521	0.008482	−1.123	0.26834
Precentral Gyrus - High > Low ED X RYGB	−0.160163	0.048306	−3.316	0.00195**
Precentral Gyrus - High > Low ED X NT	−0.122966	0.051723	−2.377	0.02231*
**18 Months**				
Precentral Gyrus - High > Low ED	0.05547	0.12408	0.447	0.659
RYGB	−0.27959	0.03064	−9.126	1.37E-09***
dWL	0.0323	0.03027	1.067	0.296
NT	0.01071	0.05258	0.204	0.84
Precentral Gyrus - High > Low ED X RYGB	−0.14382	0.16498	−0.872	0.391
Precentral Gyrus - High > Low ED X NT	0.25565	0.26797	0.954	0.349

* p<0.05

** p<0.01

*** p<0.001

## Abbreviations

BMI: Body Mass Index
CSO: Clinically severe obesity
CDC: Centers for Disease Control and Prevention
FDR: False Discovery Rate
high ED: High Energy-Dense Food
low ED: Low Energy-Dense Food
ROI: Region of Interest fMRI
RYGB: Roux-En-Y Gastric Bypass
4 mo: 4 months after intervention
18 mo: 18 months after intervention
dWL: Dietary Weight Loss
