# Supplementary material for: fMRI Food Cue Reactivity as a Predictor for BMI Change Following Roux-en-Y Gastric Bypass (RYGB) or Diet Intervention
Source: J Obes Chronic Dis. Author manuscript; Available in PMC 2022 Apr 25. (PMC9038145; doi:10.17756/jocd.2021-043)
Supplement: Supplementary [file NIHMS1793980-supplement-Supplementary.docx]

**Supplement**

| **Table 1** | | | | |
| --- | --- | --- | --- | --- |
| **Region of Interest (ROI)** | **Hem** | **X** | **Y** | **Z** |
| Frontal Operculum | R | 46 | 18 | 6 |
| Insula | R | 44 | 12 | -16 |
| Middle Cingulate Cortex | L | -2 | 10 | 42 |
| Middle Cingulate Cortex | R | 4 | 4 | 42 |
| Nucleus Accumbens | R | 2 | 6 | -10 |
| Anterior Cingulate | R | 18 | 46 | -2 |
| Cingulate Gyrus | L,R | 0 | 22 | 46 |
| Inferior Parietal Lobule | L | -32 | -40 | 50 |
| Lentiform Nucleus | R | 22 | -2 | 2 |
| Medial Frontal Gyrus | L,R | 0 | -26 | 74 |
| Middle Frontal Gyrus | R | 42 | 12 | 20 |
| Posterior Cingulate | L | -6 | -36 | -18 |
| Precentral Gyrus | L | -48 | -2 | 28 |
| Precuneus | L | -30 | -66 | 26 |
| Superior Frontal Gyrus | L,R | 6 | 18 | 66 |
